# Reprogramming T cells as an emerging treatment to slow human age-related decline in health

**DOI:** 10.3389/fmedt.2024.1384648

**Published:** 2024-04-11

**Authors:** Philip V. Peplow

**Affiliations:** Department of Anatomy, University of Otago, Dunedin, New Zealand

**Keywords:** reprogramming T cells, treatment, age-related decline, human, health

## Introduction

Human subjects are living longer due to improvements in personal and medical care. However, this is associated with an increase in age-related diseases and disorders (e.g., neurodegenerative diseases such as Alzheimer's disease, Parkinson's disease; musculoskeletal disorders such as osteoarthritis, osteoporosis, rheumatoid arthritis; metabolic disorders such as obesity, diabetes; cardiovascular disorders such as heart failure, hypertension) with increased personal and hospital costs, and many patients having to be cared for in nursing homes. A major problem in countries such as China with a very large population is that there will not be sufficient specialized facilities to care for their aging population who develop dementia and other age-related disorders. The burden of disease due to cancer in China is significant with lifestyle-related cancers including lung cancer, colorectal cancer, and breast cancer increasing and being the most prevalent cancer types in China (1). Up till now, exercise and diet have been the main interventions used to slow down the aging process, and a recent study has demonstrated that caloric restriction may slow aging in humans (2).

Senescent cells, which increase in number over time, play a role in age-related tissue decline. Genetic removal of senescent cells can reduce various age-related pathologies, including metabolic dysfunction and decreased physical fitness. Drugs have been used to remove senescent cells but require continuous administration. Reprogramming patients' disease-fighting T cells to target diseased and senescent cells may be a way for human subjects to live a healthier, better quality of life, with fewer major illnesses. Low doses of genetically modified T cells could be administered to adults to destroy senescent cells, and this would avoid undesirable side effects possibly caused by large doses.

## Helper and killer T cells

Like all white blood cells (which includes granulocytes, monocytes and lymphocytes, the latter comprising T and B cells), T cells are created in the bone marrow and mature in the thymus acquiring special markers that determine whether they will become a helper or killer T cell. A unique receptor on the surface of each cell can recognize one type of antigen on antigen-presenting cells. Helper T cells play the main role in coordinating an immune response once, for example, a virus or bacteria has been identified. Helper T cells in the thymus acquire a protein marker known as CD4, whereas T cells in the thymus that acquire a protein marker known as CD8 become cytotoxic, or killer, T cells. These T cells can recognize and destroy tumor cells.

## T cell therapy

A recent study in young mice found that genetically modified (reprogrammed) T cells erased age-related deterioration by targeting senescent cells and the mice regained their youthful characteristics while also avoiding inflammation. Giving reprogrammed T cells to aged mice caused them to rejuvenate (3). There are several reports of successfully using genetically engineered T cells to treat human cancer patients (4). Also, it has been shown that CD8 T cells can infiltrate the brain in mice and was dependent on luminal expression of major histocompatibility complex class 1 by cerebral endothelium (5). This suggests that genetically modified T cells can cross the blood brain barrier and could be used in the neuro-oncology setting (6) and possibly to treat other brain diseases.

## Production of CAR-T cells

Leukapheresis is used to obtain healthy white blood cells to treat patients by chimeric antigen receptor (CAR)-T cells. Most CAR-T cells are produced from autologous peripheral blood mononuclear cells (PBMCs), followed by T cell selection, activation, gene modification, and expansion (7). The T cells are reprogrammed by genetic modification that may involve introducing a genetic sequence through a lentiviral vector so that the T cells produce surface receptors called CARs. The CAR-T cells can now attach to specific marker proteins on the surface of targeted cells leading to their destruction. The CAR-T cells are increased in number *ex vivo* and, after the cancer patients have received chemotherapy, are administered back to the patient, with monitoring to ensure any side effects are managed appropriately (8). This technique has the potential to treat cancers, diabetes, autoimmune and inflammatory diseases.

Clinical studies with first generation CAR-T cells had poor results due to the low number of these cells existing *in vivo*. By incorporating co-stimulatory signal domains from CD28, 4-1BB, CD134 or an inducible co-stimulator into the CAR intracellular structure, the number of the CAR-T cells was significantly improved and they had promising results in cancer patients. Third-generation CARs have been developed to include not only CD3*ζ* (CD247) and one co-stimulatory domain but also an additional co-stimulatory signal (9). Fourth-generation CAR-T cells co-express some key cytokines or co-stimulatory ligands, such as IL-12, IL-15, and IL-7, or suicide genes, which significantly enhance the expansion activity of T cells. To avoid host immune rejection or graft-vs.-host disease against transplanted CAR-T cells, it has been proposed for fifth generation CAR-T cells to knock out the human leukocyte antigen (HLA) and T-cell receptor (TCR) genes of T cells obtained from healthy donors (10–12). This strategy can be used for the treatment of multiple patients as it would not require being modified depending on the patient (11).

CAR-T cells co-expressing ligand proteins of immune activating receptors, such as 4-1BB ligand (4-1BB-L) or ICOS ligand ICOS-L), to produce co-stimulatory signaling (13, 14) have been developed. CD38 CAR-T cells with CD28 signaling and 4-1BB-L co-stimulatory signaling had a potent anti-tumor effect (13).

## Commercial production of CAR-T cells

The commercial production of CAR-T cells has several limitations including the rather long time between their production and administration, with the need for bridging therapy, the requirement to cryopreserve the product, and issues with accessibility and affectability (15). Some of these limitations could be overcome by point of care CAR-T cells with shorter time from production to administration and thus not requiring cryopreservation or bridging therapy. Several technologies are being developed to increase and optimize the production of next generation CAR-T cells. Apheresis is the most widely used method in commercial CAR-T cell production protocols. Apheresis collects the mononuclear cell layer from anticoagulated whole blood, with patients being connected to a device that moves peripheral blood through a single-use disposable tubing set. The blood is separated by centrifugal force into appropriate density bands for isolation and collection of the desired cell layer. The use of automated manufacturing stations may enhance capacity and throughput without introducing significant product handling (16).

## Accessibility, cost, applicability of CAR-T cell therapy

In the USA, the FDA approved CAR-T cell therapies in 2017, and the one-time treatments has led to unprecedented response rates in patients with diffuse large B cell lymphoma and B-cell acute lymphocytic leukemia. In the UK, it was approved in 2018 for treating children and adults with lymphoma and leukaemia. In the USA, an infusion of CAR-T cell therapy costs approximately $400,000, while in the UK the tisagenlecleucel form of CAR-T, also known as Kymriah, costs around £282,000 per patient at its full list price. In Canada, the total cost of CD 19-targeted CAR-T cell therapy was estimated to be $62,500 per patient in the first year, and of patient monitoring and follow-up for five years following CAR-T cell administration estimated to be $17,160 (17). In the USA the cost of treating cancer patients with several lines of chemotherapy vs. CAR-T cell therapies is considered not to be very different. In India, CAR-T cell therapy with actalycabtagene autoleucel has been undertaken in 38 patients with lymphoma and 15 with leukemia: 26 of 38 patients with lymphoma (68%) and 11 of 15 patients with leukemia (72%) responded to the treatment. All of the patients in the leukemia group had no signs of cancer. None of the 53 patients developed the neurologic side effects that are often seen in patients treated with CAR-T cell therapies approved in the USA, and only a small proportion (5%) had a severe form of an immune-related side effect known as cytokine release syndrome. Only five patients required hospitalization because of side effects. The cost of NexCAR 19 treatment is expected to be approximately $50,000 (18). As more pharma companies and laboratories become involved with this new technology, the costs are likely to become lower as further advances and innovations take place. Some hospitals have introduced a scoring system to aid selection of patients for CAR-T cell therapy (19, 20) and maximize benefit for patients (21). By using CAR-T cell therapy to slow age-related decline in health, the number of patients requiring hospital treatment (e.g., for cancer) would be reduced leading to a substantial saving in healthcare costs. Some medical insurance companies cover the cost of CAR-T cell therapy.

At present, CAR-T cell therapy has been used for treating lymphoma and leukaemia cancer patients. Recently, it has been shown that glioblastoma, a very aggressive brain cancer, shrinks after CAR-T cell therapy (22). A team in Australia is investigating the use of CAR-T Reg cells to suppress the immune response that attacks the pancreas, causing type 1 diabetes (23). In laboratory models, CAR-T Reg cells have shown great potential for treating asthma (24), hemophilia (25) and autoimmune diseases (26).

## Future studies

Firstly, it would be important to replicate the findings of CAR-T cell therapy in young and aged mice as reported by Amor et al. (3). Normal healthy mice could be used, and then possibly to treat some mouse models of neurodegenerative disease (e.g., Alzheimer's disease, Parkinson's disease) and perform behavioral testing to see if there is an improvement in memory/learning/movement. Clinical trials could be started with healthy human subjects to examine safety and tolerability of low dose CAR-T cell therapy.

This is a personal opinion of an emerging treatment modality that could have significant impact on slowing age-related decline in human health and wellbeing, as well as reducing personal and hospital costs. Further information can be found in the cited references.
